# Dibenzyl sulfoxide

**DOI:** 10.1107/S1600536810052165

**Published:** 2010-12-18

**Authors:** Yun-Xiu Zeng, Zhi-Guang Xu, Qing-Guang Zhan, Hai-Yang Liu

**Affiliations:** a School of Chemistry and Environment, South China Normal University, Guangzhou 510006, People’s Republic of China; b Department of Chemistry, South China University of Technology, Guangzhou,510641, People’s Republic of China

## Abstract

There are two independent mol­ecules in the asymmetric unit of the title compound, C_14_H_14_OS, which have asymmetric S—C bonds [1.791 (5) and 1.804 (5) Å in one mol­ecule and 1.798 (5) and 1.804 (5) Å in the other]. The long axes of the mol­ecules are directed along the crystallographic *b* axis.

## Related literature

For related structures, see: Li *et al.* (2003 ▶); Iitaka *et al.* (1986 ▶). For the preparation, see: Shriner *et al.* (1930 ▶). For the use of sulfoxides in the separation of palladium from other platinum-group metals by solvent extraction, see: Xu *et al.* (2006 ▶).
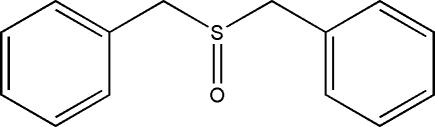


## Experimental

### 

#### Crystal data

C_14_H_14_OS
*M* *_r_* = 230.32
Orthorhombic, 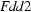

*a* = 17.882 (5) Å
*b* = 53.150 (14) Å
*c* = 10.233 (3) Å
*V* = 9726 (5) Å^3^
*Z* = 32
Mo *K*α radiation
μ = 0.24 mm^−1^
*T* = 298 K
0.36 × 0.28 × 0.15 mm

#### Data collection

Bruker APEXII CCD area-detector diffractometer
14310 measured reflections
5111 independent reflections
2563 reflections with *I* > 2σ(*I*)
*R*_int_ = 0.042

#### Refinement

*R*[*F* ^2^ > 2σ(*F* ^2^)] = 0.057
*wR*(*F* ^2^) = 0.183
*S* = 0.97
5111 reflections
289 parameters
1 restraint
H-atom parameters constrained
Δρ_max_ = 0.56 e Å^−3^
Δρ_min_ = −0.19 e Å^−3^
Absolute structure: Flack (1983 ▶), 1074 Friedel pairs
Flack parameter: 0.00 (12)

### 

Data collection: *APEX2* (Bruker, 2005 ▶); cell refinement: *SAINT* (Bruker, 2005 ▶); data reduction: *SAINT*; program(s) used to solve structure: *SHELXS97* (Sheldrick, 2008 ▶); program(s) used to refine structure: *SHELXL97* (Sheldrick, 2008 ▶); molecular graphics: *ORTEP-3 for Windows* (Farrugia, 1997 ▶); software used to prepare material for publication: *SHELXL97* and *PLATON* (Spek, 2009 ▶).

## Supplementary Material

Crystal structure: contains datablocks I, global. DOI: 10.1107/S1600536810052165/jh2245sup1.cif

Structure factors: contains datablocks I. DOI: 10.1107/S1600536810052165/jh2245Isup2.hkl

Additional supplementary materials:  crystallographic information; 3D view; checkCIF report

## Figures and Tables

**Fig. 1.**
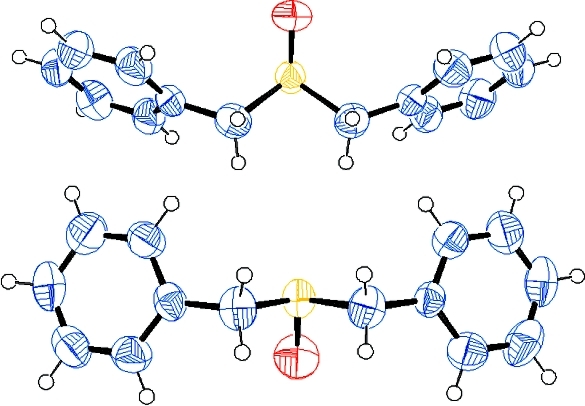
Molecule structure of with displacement ellipsoids drawn at the 50% probability level.

## supplementary crystallographic information

### Comment

Sulfoxides have been widely used in the separation of palladium from other platinum-group metals by solvent extraction (Xu *et al.*, 2006).A similar disulfoxide ligand 1,6-bis(benzylsulfinyl)hexane and its Copper(II) and Cadmium(II) dimeric complexes were obtained (Li *et al.*,2003). Crystals of dibenzyl sulfoxide show two independent molecules in the unit. There are asymmetry S—C bonds in a same molecule. The long axe of the crystals is directed along the *b* axis.

### Experimental

The title compound was prepared refering to the literature method (Shriner *et al.*, 1930) with little modification. Sodium sulfide(99%, 0.312 g, 0.0040 mol) and benzylchloride (1.000 g, 0.0079 mol) were dissolved in anhydrous ethanol (50 ml) at 70°C, and then was stirred over 1 h. The solution was extracted with CH_2_Cl_2_ after addition 400 ml of water. Dibenzyl sulfide(0.736 g, 0.0034 mol) was obtained after evaporation of CH_2_Cl_2_. Yield: 86%. Hydrogen peroxide (30%, 0.0028 mol) was added dropwise to a solution of dibenzyl sulfide (0.600 g, 0.0028 mol) in acetic acid (60 ml) on ice bath with a vigorously stir for 1 h. 500 ml of water was added. The solution was extracted with CH_2_Cl_2_, and the product of dibenzyl sulfoxide(0.552 g, 0.0024 mol) was obtained after evaporation of CH_2_Cl_2_. Yield: 86%. It was characterized by recording its infrared and NMR spectra. White single crystals of the title compound were obtained by slow evaporation of its mixed solution including n-hexane and dichloromethane.)

### Refinement

(All H atoms were placed in calculated positions and subsequently constrained to ride on their parent atoms, with C–H distances of 0.93 Å (C-aromatic) and 0.97 Å (*C*-methyl). The *U*_iso_(H) values were set at 1.2 *U*_eq_(C aromatic) and 1.5 *U*_eq_(C methylene).)

### Crystal data

**Table:**

C_14_H_14_OS	*F*(000) = 3904
*M**_r_* = 230.32	*D*_x_ = 1.258 Mg m^−^^3^
Orthorhombic, *F**d**d*2	Mo *K*α radiation, λ = 0.71073 Å
Hall symbol: F 2 -2d	Cell parameters from 2370 reflections
*a* = 17.882 (5) Å	θ = 2.3–23.7°
*b* = 53.150 (14) Å	µ = 0.24 mm^−^^1^
*c* = 10.233 (3) Å	*T* = 298 K
*V* = 9726 (5) Å^3^	Block, white
*Z* = 32	0.36 × 0.28 × 0.15 mm

### Data collection

**Table:**

Bruker APEXII CCD area-detector diffractometer	2563 reflections with *I* > 2σ(*I*)
Radiation source: fine-focus sealed tube	*R*_int_ = 0.042
graphite	θ_max_ = 28.3°, θ_min_ = 2.3°
phi and ω scans	*h* = −23→22
14310 measured reflections	*k* = −56→69
5111 independent reflections	*l* = −11→13

### Refinement

**Table:**

Refinement on *F*^2^	Secondary atom site location: difference Fourier map
Least-squares matrix: full	Hydrogen site location: inferred from neighbouring sites
*R*[*F*^2^ > 2σ(*F*^2^)] = 0.057	H-atom parameters constrained
*wR*(*F*^2^) = 0.183	*w* = 1/[σ^2^(*F*_o_^2^) + (0.0957*P*)^2^] where *P* = (*F*_o_^2^ + 2*F*_c_^2^)/3
*S* = 0.97	(Δ/σ)_max_ = 0.061
5111 reflections	Δρ_max_ = 0.56 e Å^−^^3^
289 parameters	Δρ_min_ = −0.19 e Å^−^^3^
1 restraint	Absolute structure: Flack (1983), 1074 Friedel pairs
Primary atom site location: structure-invariant direct methods	Flack parameter: 0.00 (12)

### Special details

**Table:**

Geometry. All e.s.d.'s (except the e.s.d. in the dihedral angle between two l.s. planes)are estimated using the full covariance matrix. The cell e.s.d.'s are takeninto account individually in the estimation of e.s.d.'s in distances, anglesand torsion angles; correlations between e.s.d.'s in cell parameters are onlyused when they are defined by crystal symmetry. An approximate (isotropic)treatment of cell e.s.d.'s is used for estimating e.s.d.'s involving l.s. planes.
Refinement. Refinement of *F*^2^ against ALL reflections. The weighted *R*-factor *wR* and goodness of fit *S* are based on *F*^2^, conventional *R*-factors *R* are based on *F*, with *F* set to zero for negative *F*^2^. The threshold expression of *F*^2^ > σ(*F*^2^) is used only for calculating *R*-factors(gt) *etc*. and is not relevant to the choice of reflections for refinement. *R*-factors based on *F*^2^ are statistically about twice as large as those based on *F*, and *R*- factors based on ALL data will be even larger.

### Fractional atomic coordinates and isotropic or equivalent isotropic displacement parameters (Å^2^)

**Table:**

	*x*	*y*	*z*	*U*_iso_*/*U*_eq_	
S1	0.40616 (7)	0.12486 (3)	0.42542 (10)	0.0541 (3)	
C6	0.1374 (2)	0.07435 (9)	0.7186 (5)	0.0511 (13)	
C9	0.1385 (3)	0.17549 (9)	0.7183 (5)	0.0547 (13)	
C7	0.1315 (3)	0.10024 (9)	0.7775 (5)	0.0657 (13)	
H7A	0.1713	0.1024	0.8410	0.079*	
H7B	0.0842	0.1017	0.8233	0.079*	
C8	0.1322 (3)	0.14959 (9)	0.7780 (5)	0.0637 (13)	
H8A	0.0850	0.1483	0.8241	0.076*	
H8B	0.1720	0.1474	0.8412	0.076*	
C4	0.0819 (3)	0.03513 (11)	0.6580 (6)	0.0878 (18)	
H4	0.0400	0.0248	0.6511	0.105*	
C13	0.2163 (3)	0.20854 (11)	0.6307 (6)	0.0814 (16)	
H13	0.2630	0.2144	0.6045	0.098*	
C1	0.2051 (3)	0.06488 (10)	0.6809 (6)	0.0693 (13)	
H1	0.2475	0.0749	0.6894	0.083*	
C2	0.2122 (3)	0.04126 (10)	0.6312 (6)	0.0796 (16)	
H2	0.2590	0.0355	0.6050	0.096*	
C5	0.0754 (3)	0.05917 (11)	0.7057 (6)	0.0747 (15)	
H5	0.0286	0.0653	0.7297	0.090*	
C3	0.1526 (4)	0.02621 (10)	0.6196 (6)	0.0782 (17)	
H3	0.1578	0.0100	0.5865	0.094*	
C11	0.0846 (3)	0.21459 (11)	0.6523 (6)	0.0790 (17)	
H11	0.0425	0.2247	0.6414	0.095*	
C14	0.2086 (3)	0.18479 (9)	0.6809 (6)	0.0697 (14)	
H14	0.2507	0.1747	0.6903	0.084*	
C12	0.1542 (3)	0.22363 (10)	0.6195 (6)	0.0774 (17)	
H12	0.1593	0.2401	0.5896	0.093*	
C10	0.0773 (3)	0.19068 (10)	0.7010 (5)	0.0707 (15)	
H10	0.0301	0.1847	0.7227	0.085*	
C17	0.4166 (3)	0.02607 (10)	0.3893 (7)	0.0738 (16)	
H17	0.4294	0.0096	0.3678	0.089*	
C26	0.4207 (3)	0.22365 (10)	0.3836 (7)	0.0742 (16)	
H26	0.4354	0.2399	0.3609	0.089*	
C15	0.4119 (3)	0.05920 (10)	0.5446 (6)	0.0726 (15)	
H15	0.4225	0.0653	0.6277	0.087*	
C22	0.3498 (3)	0.14955 (10)	0.4925 (6)	0.0674 (14)	
H22A	0.2987	0.1474	0.4630	0.081*	
H22B	0.3501	0.1482	0.5870	0.081*	
C24	0.3599 (3)	0.18525 (10)	0.3314 (6)	0.0695 (15)	
H24	0.3334	0.1755	0.2718	0.083*	
C28	0.4160 (3)	0.19012 (10)	0.5414 (6)	0.0737 (15)	
H28	0.4283	0.1839	0.6236	0.088*	
C19	0.3607 (3)	0.06475 (10)	0.3319 (6)	0.0697 (14)	
H19	0.3366	0.0749	0.2710	0.084*	
C20	0.3754 (3)	0.07420 (9)	0.4574 (5)	0.0525 (13)	
C16	0.4335 (3)	0.03512 (10)	0.5121 (6)	0.0823 (17)	
H16	0.4589	0.0251	0.5720	0.099*	
C27	0.4374 (3)	0.21400 (10)	0.5059 (7)	0.0844 (18)	
H27	0.4636	0.2239	0.5652	0.101*	
C21	0.3494 (3)	0.10017 (9)	0.4944 (6)	0.0655 (14)	
H21A	0.3498	0.1017	0.5888	0.079*	
H21B	0.2982	0.1023	0.4652	0.079*	
C23	0.3763 (3)	0.17547 (9)	0.4545 (5)	0.0537 (13)	
C25	0.3822 (3)	0.20898 (11)	0.2968 (6)	0.0818 (16)	
H25	0.3711	0.2151	0.2140	0.098*	
C18	0.3813 (3)	0.04094 (12)	0.2979 (6)	0.0823 (16)	
H18	0.3716	0.0348	0.2144	0.099*	
O1	0.47890 (18)	0.12488 (7)	0.4984 (4)	0.0831 (11)	
O2	0.0644 (2)	0.12505 (7)	0.5840 (4)	0.0850 (11)	
S2	0.13762 (7)	0.12488 (3)	0.65757 (9)	0.0548 (3)	

### Atomic displacement parameters (Å^2^)

**Table:**

	*U*^11^	*U*^22^	*U*^33^	*U*^12^	*U*^13^	*U*^23^
S1	0.0557 (6)	0.0490 (6)	0.0575 (8)	0.0010 (6)	0.0076 (5)	0.0000 (7)
C6	0.057 (3)	0.044 (3)	0.053 (3)	−0.003 (2)	−0.002 (2)	0.007 (2)
C9	0.064 (3)	0.048 (3)	0.052 (3)	0.005 (2)	−0.010 (2)	−0.010 (3)
C7	0.080 (3)	0.064 (3)	0.053 (3)	−0.005 (3)	−0.002 (3)	0.006 (3)
C8	0.083 (3)	0.061 (3)	0.047 (3)	0.000 (2)	−0.004 (3)	−0.006 (2)
C4	0.100 (4)	0.077 (4)	0.086 (4)	−0.037 (3)	−0.024 (4)	0.003 (4)
C13	0.082 (4)	0.076 (4)	0.087 (4)	−0.009 (3)	0.000 (3)	−0.002 (3)
C1	0.064 (3)	0.066 (3)	0.078 (4)	−0.005 (2)	−0.002 (3)	0.009 (3)
C2	0.088 (4)	0.066 (4)	0.085 (4)	0.009 (3)	0.007 (3)	0.007 (3)
C5	0.063 (3)	0.079 (4)	0.082 (4)	−0.002 (3)	−0.017 (3)	0.006 (3)
C3	0.121 (5)	0.048 (4)	0.066 (4)	−0.007 (3)	−0.004 (4)	−0.003 (3)
C11	0.079 (4)	0.070 (4)	0.088 (4)	0.022 (3)	−0.019 (4)	−0.015 (3)
C14	0.061 (3)	0.068 (3)	0.080 (4)	0.009 (2)	−0.007 (3)	−0.009 (3)
C12	0.114 (5)	0.050 (4)	0.068 (4)	0.005 (3)	−0.006 (3)	−0.007 (3)
C10	0.057 (3)	0.077 (4)	0.079 (4)	0.004 (2)	−0.008 (3)	−0.011 (3)
C17	0.073 (3)	0.054 (4)	0.094 (5)	0.002 (3)	0.010 (3)	0.001 (3)
C26	0.074 (3)	0.051 (4)	0.098 (5)	0.002 (3)	0.009 (3)	−0.005 (3)
C15	0.072 (3)	0.080 (4)	0.065 (3)	−0.001 (3)	−0.014 (3)	0.008 (3)
C22	0.061 (3)	0.066 (4)	0.074 (4)	0.005 (2)	0.014 (3)	−0.008 (3)
C24	0.072 (3)	0.059 (3)	0.077 (4)	0.004 (2)	−0.007 (3)	−0.011 (3)
C28	0.074 (3)	0.069 (4)	0.078 (4)	0.011 (3)	−0.014 (3)	−0.014 (3)
C19	0.075 (3)	0.064 (3)	0.070 (4)	−0.002 (3)	−0.013 (3)	0.009 (3)
C20	0.051 (3)	0.048 (3)	0.058 (3)	−0.005 (2)	0.004 (2)	0.006 (3)
C16	0.081 (4)	0.072 (4)	0.094 (5)	0.008 (3)	−0.010 (4)	0.021 (3)
C27	0.074 (4)	0.069 (4)	0.110 (5)	−0.001 (3)	−0.016 (4)	−0.033 (3)
C21	0.063 (3)	0.063 (3)	0.071 (4)	−0.002 (2)	0.013 (3)	0.001 (3)
C23	0.052 (3)	0.046 (3)	0.063 (3)	0.007 (2)	0.006 (2)	−0.007 (3)
C25	0.091 (4)	0.074 (4)	0.080 (4)	0.015 (3)	0.002 (3)	0.005 (3)
C18	0.096 (4)	0.077 (4)	0.073 (4)	−0.008 (3)	−0.003 (3)	−0.006 (3)
O1	0.0486 (17)	0.083 (2)	0.117 (3)	0.0017 (15)	−0.011 (2)	−0.004 (3)
O2	0.088 (3)	0.099 (3)	0.067 (2)	0.001 (2)	−0.034 (2)	0.003 (2)
S2	0.0703 (8)	0.0508 (6)	0.0434 (6)	0.0005 (6)	0.0020 (6)	−0.0014 (7)

### Geometric parameters (Å, °)

**Table:**

S1—O1	1.500 (3)	C10—H10	0.9300
S1—C22	1.791 (5)	C17—C18	1.378 (8)
S1—C21	1.804 (5)	C17—C16	1.379 (8)
C6—C1	1.367 (6)	C17—H17	0.9300
C6—C5	1.378 (6)	C26—C25	1.368 (8)
C6—C7	1.506 (6)	C26—C27	1.385 (8)
C9—C10	1.371 (6)	C26—H26	0.9300
C9—C14	1.401 (6)	C15—C20	1.363 (7)
C9—C8	1.510 (6)	C15—C16	1.377 (7)
C7—S2	1.798 (5)	C15—H15	0.9300
C7—H7A	0.9700	C22—C23	1.508 (7)
C7—H7B	0.9700	C22—H22A	0.9700
C8—S2	1.804 (5)	C22—H22B	0.9700
C8—H8A	0.9700	C24—C25	1.369 (7)
C8—H8B	0.9700	C24—C23	1.395 (7)
C4—C5	1.373 (8)	C24—H24	0.9300
C4—C3	1.405 (8)	C28—C27	1.374 (8)
C4—H4	0.9300	C28—C23	1.379 (7)
C13—C14	1.370 (7)	C28—H28	0.9300
C13—C12	1.375 (7)	C19—C18	1.363 (7)
C13—H13	0.9300	C19—C20	1.404 (7)
C1—C2	1.360 (7)	C19—H19	0.9300
C1—H1	0.9300	C20—C21	1.505 (6)
C2—C3	1.338 (7)	C16—H16	0.9300
C2—H2	0.9300	C27—H27	0.9300
C5—H5	0.9300	C21—H21A	0.9700
C3—H3	0.9300	C21—H21B	0.9700
C11—C10	1.371 (8)	C25—H25	0.9300
C11—C12	1.376 (8)	C18—H18	0.9300
C11—H11	0.9300	O2—S2	1.511 (3)
C14—H14	0.9300	S2—C7	1.798 (5)
C12—H12	0.9300	S2—C8	1.804 (5)
			
O1—S1—C22	107.2 (2)	C18—C17—C16	121.3 (6)
O1—S1—C21	107.1 (2)	C18—C17—H17	119.4
C22—S1—C21	93.8 (2)	C16—C17—H17	119.4
C1—C6—C5	118.1 (5)	C25—C26—C27	119.0 (5)
C1—C6—C7	120.8 (5)	C25—C26—H26	120.5
C5—C6—C7	121.1 (5)	C27—C26—H26	120.5
C10—C9—C14	118.1 (5)	C20—C15—C16	121.3 (6)
C10—C9—C8	122.0 (5)	C20—C15—H15	119.3
C14—C9—C8	119.9 (4)	C16—C15—H15	119.3
C6—C7—S2	112.8 (4)	C23—C22—S1	113.2 (3)
C6—C7—H7A	109.0	C23—C22—H22A	108.9
S2—C7—H7A	109.0	S1—C22—H22A	108.9
C6—C7—H7B	109.0	C23—C22—H22B	108.9
S2—C7—H7B	109.0	S1—C22—H22B	108.9
H7A—C7—H7B	107.8	H22A—C22—H22B	107.7
C9—C8—S2	112.6 (4)	C25—C24—C23	121.0 (6)
C9—C8—H8A	109.1	C25—C24—H24	119.5
S2—C8—H8A	109.1	C23—C24—H24	119.5
C9—C8—H8B	109.1	C27—C28—C23	119.6 (6)
S2—C8—H8B	109.1	C27—C28—H28	120.2
H8A—C8—H8B	107.8	C23—C28—H28	120.2
C5—C4—C3	119.4 (5)	C18—C19—C20	121.0 (5)
C5—C4—H4	120.3	C18—C19—H19	119.5
C3—C4—H4	120.3	C20—C19—H19	119.5
C14—C13—C12	119.2 (5)	C15—C20—C19	118.6 (5)
C14—C13—H13	120.4	C15—C20—C21	121.3 (5)
C12—C13—H13	120.4	C19—C20—C21	120.0 (5)
C2—C1—C6	121.9 (5)	C15—C16—C17	118.9 (5)
C2—C1—H1	119.1	C15—C16—H16	120.6
C6—C1—H1	119.1	C17—C16—H16	120.6
C3—C2—C1	120.7 (5)	C28—C27—C26	121.4 (5)
C3—C2—H2	119.7	C28—C27—H27	119.3
C1—C2—H2	119.7	C26—C27—H27	119.3
C4—C5—C6	120.7 (5)	C20—C21—S1	113.2 (3)
C4—C5—H5	119.7	C20—C21—H21A	108.9
C6—C5—H5	119.7	S1—C21—H21A	108.9
C2—C3—C4	119.3 (5)	C20—C21—H21B	108.9
C2—C3—H3	120.3	S1—C21—H21B	108.9
C4—C3—H3	120.3	H21A—C21—H21B	107.7
C10—C11—C12	119.8 (5)	C28—C23—C24	118.7 (5)
C10—C11—H11	120.1	C28—C23—C22	120.8 (5)
C12—C11—H11	120.1	C24—C23—C22	120.5 (5)
C13—C14—C9	121.1 (5)	C26—C25—C24	120.2 (6)
C13—C14—H14	119.4	C26—C25—H25	119.9
C9—C14—H14	119.4	C24—C25—H25	119.9
C13—C12—C11	120.5 (6)	C19—C18—C17	118.9 (6)
C13—C12—H12	119.8	C19—C18—H18	120.6
C11—C12—H12	119.8	C17—C18—H18	120.6
C11—C10—C9	121.1 (5)	O2—S2—C7	107.0 (2)
C11—C10—H10	119.4	O2—S2—C8	106.8 (2)
C9—C10—H10	119.4	C7—S2—C8	93.5 (2)
			
C1—C6—C7—S2	77.1 (6)	C18—C19—C20—C15	1.0 (8)
C5—C6—C7—S2	−105.4 (5)	C18—C19—C20—C21	−177.4 (5)
C10—C9—C8—S2	103.3 (5)	C20—C15—C16—C17	−0.9 (8)
C14—C9—C8—S2	−77.8 (6)	C18—C17—C16—C15	2.3 (9)
C5—C6—C1—C2	0.4 (8)	C23—C28—C27—C26	−0.8 (8)
C7—C6—C1—C2	177.9 (5)	C25—C26—C27—C28	−0.1 (9)
C6—C1—C2—C3	−1.2 (9)	C15—C20—C21—S1	105.3 (5)
C3—C4—C5—C6	−1.2 (9)	C19—C20—C21—S1	−76.3 (6)
C1—C6—C5—C4	0.8 (8)	O1—S1—C21—C20	−72.8 (5)
C7—C6—C5—C4	−176.7 (5)	C22—S1—C21—C20	177.8 (4)
C1—C2—C3—C4	0.7 (9)	C27—C28—C23—C24	1.0 (8)
C5—C4—C3—C2	0.5 (9)	C27—C28—C23—C22	−177.9 (4)
C12—C13—C14—C9	1.4 (9)	C25—C24—C23—C28	−0.4 (8)
C10—C9—C14—C13	1.2 (8)	C25—C24—C23—C22	178.5 (5)
C8—C9—C14—C13	−177.7 (5)	S1—C22—C23—C28	−102.4 (5)
C14—C13—C12—C11	−3.2 (9)	S1—C22—C23—C24	78.7 (6)
C10—C11—C12—C13	2.4 (9)	C27—C26—C25—C24	0.7 (9)
C12—C11—C10—C9	0.2 (8)	C23—C24—C25—C26	−0.5 (8)
C14—C9—C10—C11	−1.9 (8)	C20—C19—C18—C17	0.3 (8)
C8—C9—C10—C11	176.9 (5)	C16—C17—C18—C19	−2.0 (9)
O1—S1—C22—C23	72.7 (5)	C6—C7—S2—O2	73.7 (4)
C21—S1—C22—C23	−178.1 (5)	C6—C7—S2—C8	−177.5 (4)
C16—C15—C20—C19	−0.7 (8)	C9—C8—S2—O2	−73.3 (4)
C16—C15—C20—C21	177.7 (5)	C9—C8—S2—C7	177.8 (4)
